# Exploring Additive, Synergistic or Antagonistic Effects of Natural Plant Extracts on In Vitro Beef Feedlot-Type Rumen Microbial Fermentation Conditions

**DOI:** 10.3390/ani10010173

**Published:** 2020-01-20

**Authors:** Ignacio Fandiño, Gonzalo Fernandez-Turren, Alfred Ferret, Diego Moya, Lorena Castillejos, Sergio Calsamiglia

**Affiliations:** 1 Departamento de Ciència Animal i dels Aliments, Universitat Autònoma de Barcelona, Servicio de Nutrición y Bienestar Animal (SNiBA), 08193 Bellaterra, Spain; alfred.ferret@uab.cat (A.F.); diego.moya@usask.ca (D.M.); Lorena.castillejos@uab.cat (L.C.); 2 Departamento de Producción Animal, Instituto de Producción Animal, Facultad de Veterinaria, Universidad de la República, Ruta 1 km 42, CP 80100 San José, Uruguay; gonzalofernandezt@gmail.com

**Keywords:** essential oils, rumen microbial fermentation, synergies

## Abstract

**Simple Summary:**

Essential oils (EO) can be used as natural alternatives to in-feed antibiotics. Most EO products in the market are based on a combination of EO or their active molecules but prove of additivity or synergy is lacking. The effect of six EO (tea tree oil—TeTr, oregano oil—Ore, clove bud oil—Clo, thyme oil—Thy, rosemary oil—Ros and sage oil—Sag) and different mixes on in vitro microbial fermentation profile of a feedlot beef cattle type fermentation were evaluated for their additive, synergistic or antagonistic effects. Mixing TeTr with Thy, Ore or Thy + Ore modified rumen microbial fermentation profile, but the size of the effect was similar to that obtained with TeTr alone, suggesting that the effects were not additive. When Thy, Ore or Thy + Ore were mixed with Clo, most effects on rumen fermentation profile disappeared even when TeTr was part of the mix, suggesting an antagonistic interaction of Clo with Thy and Ore. Results do not support the hypothesis of additivity among the EO tested, and antagonistic effects may occur among some of them, at least in a low pH, beef-type fermentation conditions.

**Abstract:**

Six Essential oils (EO) (tea tree oil—TeTr, oregano oil—Ore, clove bud oil—Clo, thyme oil—Thy, rosemary oil—Ros, and sage oil—Sag) in Experiment 1; and different combinations of selected oils in Experiment 2, were evaluate at four doses in an in vitro microbial fermentation system using ruminal fluid from beef cattle fed a 10:90 straw: Concentrate diet. In Experiment 1, TeTr, Ore, Clo and Thy improved rumen fermentation profile in a direction consistent with better feed utilization. In Experiment 2, TeTr mixed with Thy, Ore, Thy + Ore or Clo at 200 and 400 mg/L increased the molar proportion of propionate and decreased that of acetate, and the acetate to propionate ratio. However, the size of the effect was similar to that obtained with TeTr alone, suggesting that effects were not additive. When Thy, Ore or Thy + Ore where mixed with Clo, most effects on rumen fermentation profile disappeared, suggesting an antagonistic interaction of Clo with Thy and Ore. Results do not support the hypothesis of additivity among the EO tested, and antagonistic effects of Clo mixed with Thy or Ore were demonstrated at least in a low pH, beef-type fermentation conditions.

## 1. Introduction

Essential oils (EO) are aromatic oily liquids obtained from plants that can be used as natural alternatives to in-feed antibiotics [1,2]. Many different plant EO and their active components have been tested for their effects on ruminal microbial fermentation profile [3,4,5]. Because of the diverse mechanisms of action and effects, Calsamiglia et al. [1] suggested that the wise selection and combination of different EO may enhance ruminal fermentation. When combinations of EO are used, the effect may be additive, synergistic or antagonistic. Most EO products for ruminants in the market are based on combination of different EO or their active molecules (i.e., CRINA-Ruminants^®^, DSM, Switzerland; AGOLIN^®^, Agolin Sa, Switzerland, XTRACT^®^, Pancosma, Switzerland), but proof of additivity or synergy is lacking. Some studies attributed additive and synergistic effects to phenolic and alcohol compounds. In general, compounds with similar structures exhibit additive rather than synergistic effects. Antagonistic effects have been attributed to the interaction between non-oxygenated and oxygenated monoterpene hydrocarbons [6]. All these interactions have been demonstrated in cosmetic and food industry [6,7,8]. However, it is surprising that no studies have been specifically designed to prove additivity or synergies of different EO on rumen microbial fermentation. The mechanism of action of most EO is mediated through their interaction with the cell membrane, and this interaction is dependent on the fermentation conditions like the source of rumen fluid, the substrate of fermentation and pH [1,5]. The additive, synergistic or antagonistic effects in EO mixes may also be dependent on these fermentation conditions. While most research in vitro has been conducted using rumen fluid from dairy cattle, forage as a main fermentation substrate and pH above 6.5, research in high concentrate feedlot-type beef fermentation conditions is more limited.

We hypothesize that the combination of different EO, in particular simple phenolic-type molecules of the family of monoterpenoids and phenylpropanes, will result in additive or synergistic effects. The objective of the present study was to evaluate the effects of different doses of six EO and the additive, synergistic or antagonistic effects of their combinations under feedlot beet-type ruminal microbial fermentation conditions in vitro. 

## 2. Materials and Methods

### 2.1. Experimental Protocol and Treatments 

The research protocol was approved by Campus Laboratory Animal Care Committee of the Universitat Autónoma of Barcelona (Spain) (reference CEAAH 00604). The effects of six EO and their combinations were evaluated in an in vitro batch fermentation system. Rumen fluid was obtained from four beef heifers fed a 10:90 barley straw:concentrate diet (161 g/kgcrude protein, 321 g/kg neutral detergent fiber, 168 g/kg acid detergent fiber on a dry matter basis) designed to meet or exceed nutrient recommendations [9] of growing beef cattle. The concentrate consisted of (DM basis) ground barley grain (444 g/kg), corn gluten feed (155 g/kg), ground corn grain (133 g/kg), soybean meal (72 g/kg), sunflower meal (72 g/kg), ground wheat grain (69 g/kg), palm kernel (52 g/kg), vitamin A (4600 IU/kg), vitamin D (450 IU/kg), vitamin E (7.5 mg/kg), zinc (10.5 mg/kg), iron (6 mg/kg), manganese (1.2 mg/kg), copper (0.75 mg/kg), cobalt (0.15 mg/kg), iodine (0.11 mg/kg) and selenium (0.08 mg/kg). Rumen fluid was strained through two layers of cheesecloth and mixed in a 1:1 proportion with a phosphate-bicarbonate buffer [10]. The pH of the buffered fluid was adjusted to 6.20 with hydrochloric acid. Incubations were conducted in a 75 ml tubes containing 50 ml of the diluted rumen fluid and 0.5 g of the same diet fed to the donor animals. The diet was ground through a 2 mm screen, and each tube was gassed with CO_2_ before sealing with rubber corks with a gas release valve. Incubations were conducted in a water bath at 39 °C for 24 h in two consecutive periods with triplicates within period.

In Experiment 1, four doses of different EO (10, 50, 200 and 400 mg/L of the culture fluid) were evaluated. Most EO have shown to be effective at doses around 200–400 mg/L [1,3,5]. Doses were selected based on this evidence and at lower doses to show potential additivity and synergies of effects. Treatments were a negative control without EO (CTR), a positive control with Monensin at 10 mg/L (M5273 Sigma-Aldrich Chemical, St Louis, MO, USA) and six different phenolic-derived monoterpene or phenylpropane EO, of which two were oxygenated and four non-oxygenated: 1) Tea tree oil (*Melaleuca alternifolia*; TeTr; containing 42% terpinen-4-ol as active component), Oregano oil (*Origanum vulgare*; Ore; containing 53% carvacrol as active components), Clove bud oil (*Syzygium aromaticum*; Clo; containing 82% eugenol as main active component), Thyme oil (*Thymus vulgaris*; Thy; containing 47% thymol as main active component), Rosemary oil (*Rosmarinus officinalis*; Ros; containing 61% 1,8-cineole as main active component) and Sage oil (*Salvia officinalis*; Sag; containing 65% thujone as main active component). In Experiment 2, four EO selected from Experiment 1 (TeTr, Thy, Ore and Clo) were used in 13 different combinations at four concentrations (10, 50, 200 and 400 mg/L of the culture fluid; Table 1) to test additive, synergistic or antagonist effects.

### 2.2. Sample Collection and Chemical Analyses 

After 24 h, the fermented fluid pH was measured immediately (Model 507; Crison Instruments, Alella, Barcelona, Spain) and liquid samples were withdrawn from each tube to analyze ammonia-N and volatile fatty acid (VFA) concentrations. Samples for ammonia-N analysis (5 mL of fermentation fluid preserved with 1 mL of a 50 g/L orthophosphoric acid solution) were centrifuged at 15,000× *g* for 15 min at 4 °C, and the supernatant was analyzed for ammonia-N by colorimetry [11]. Samples for VFA analysis were prepared as described by Jouany [12]. Samples of fermented fluid (5 mL) were preserved with 1 mL of a solution made of 2 g/L of mercuric chloride, 35 g/L orthophosphoric acid and 2 g/L of 4-methylvaleric acid as an internal standard, and frozen. After thawing, samples were centrifuged at 3000× *g* for 30 min, and the supernatant fraction was analyzed by gas chromatography (Model 6890; Hewlett Packard, Palo Alto, CA, USA) using a polyethylene glycol nitroterephthalic acid-treated capillary column (BP21; SGE, Europe Ltd., Buckinghamshire, UK) at 275 °C in the injector and a gas flow rate of 29.9 mL/min.

### 2.3. Statistical Analyses

Results of the batch fermentation experiment were analyzed as a randomized complete block design using the PROC MIXED procedure (SAS, version 9.4, SAS Institute Inc., Cary, NC, USA), where the essential oils or mixes were the fixed effect and the period was considered a random effect. Because the objective was to identify the lowest dose of EO at which effects were observed, the Dunnett test was used to identify significant differences (*p* < 0.05). When required, the synergistic, additive or antagonistic effects of mixes were evaluated comparing the relative response of the mixes vs. the corresponding effects of the main essential oil using a paired t-test at a significant level of *p* < 0.05.

## 3. Results

Because of the large number of treatments tested, two runs in Experiment 1, and six runs in Experiment 2 were conducted. To account for possible run-to-run variation, a negative (no additive) control was used, and results are reported as percent change compared with this negative control (Table 2 and Table 3).

### 3.1. Experiment 1 (Individual Essential Oils)

Results from Experiment 1 are shown in Table 2. Monensin was used as a positive control and always increased (*p* < 0.05) the proportion of propionate and decreased (*p* < 0.05) the molar proportion of acetate and the acetate to propionate (A:P) ratio. In most cases, monensin also reduced the molar proportion of butyrate and brach-chain VFA (BCVFA), and the concentration of ammonia-N when compared with control. 

None of the EO treatments affected rumen fermentation profile at 10 mg/L compared with control. Tea tree oil at 50 and 200 mg/L decreased (*p* < 0.05) the molar proportion of acetate (−9 to −15%) and the A:P ratio (−22 to −36%), and increased (*p* < 0.05) the molar proportion of propionate (+18 to +34%) and butyrate (+14 to +21%). At 400 mg/L TeTr only increased (*p* < 0.05) the molar proportion of butyrate (+14%) compared with control. Oregano oil at 50 and 200 mg/L decreased (*p* < 0.05) the molar proportion of acetate (−9 to −11%) and the A:P ratio (−26 to −9%) and increased (*p* < 0.05) the molar proportion of propionate (+24 to +25%) and the molar proportion of butyrate (+12 to +16%), but at 400 mg/L increased (*p* < 0.05) the molar proportion of butyrate (+17%) and the pH (+2%) compared with control. Clove bud oil at 50 mg/L had no effects on rumen microbial fermentation, but at 200 and 400 mg/L increased (*p* < 0.05) the molar proportion of butyrate (+10 to +18%), and at 400 mg/L, decreased (*p* < 0.05) the molar proportion of propionate (−13%), and increased (*p* < 0.05) the A:P ratio (+15%) and the pH (+2%) compared with control. Thyme oil at 50 and 200 decreased (*p* < 0.05) the molar proportion of acetate (−6%) and the A:P ratio (−25 to −28%), and increased (*p* < 0.05) the molar proportion of propionate (+24 to +30%). At 200 and 400 mg/L Thy decreased (*p* < 0.05) the molar proportion of butyrate (−15 to −21%), but only at 400 mg/L decreased (*p* < 0.05) the total VFA concentration (−4%), the BCVFA concentration (−16%) and the concentration of ammonia-N (−17%), and increased (*p* < 0.05) the molar proportion of acetate (+5%). Rosemary oil at 50 mg/L had no effect on rumen microbial fermentation, but at 200 and 400 mg/L decreased (*p* < 0.05) the molar proportion of butyrate (−14 to −19%) and at 400 mg/L increased (*p* < 0.05) the molar proportion of acetate (−4%) and decreased the total VFA concentration (−5%) compared with control. Sage oil at 50 mg/L had no effect on rumen microbial fermentation, but at 200 mg/L decreased (*p* < 0.05) the molar proportion of butyrate (−17%) and at 400 mg/L, increased (*p* < 0.05) the molar proportion of propionate (+12%), reduced (*p* < 0.05) the A:P ratio (−13%) and the concentration of ammonia-N (−16%), and tended to reduce (*p* < 0.10) the total VFA concentration (−4%) compared with control.

### 3.2. Experiment 2 (Combination of Essential Oils)

Treatments 7 to 13 had no effect on rumen fermentation profile and are not reported. The effects of treatments 1 to 6 are shown in Table 3. Monensin reduced (*p* < 0.05) the molar proportion of acetate, the molar proportion of butyrate, the BCVFA concentrations, the A:P ratio and the concentration of ammonia-N, and increased (*p* < 0.05) the molar proportion of propionate compared with control. At 10 and 50 mg/L, none of the treatments had effects on rumen fermentation profile compared with control. 

Treatment 1 at 200 and 400 mg/L decreased (*p* < 0.05) the molar proportion of acetate (−5 to −6%) and the A:P ratio (−16 to −19%), and increased (*p* < 0.05) the molar proportion of propionate (+12 to +16%). Treatment 1 at 400 mg/L tended to reduce (*p* < 0.10) the pH compared with control Treatment 2 only at 400 mg/L decreased (*p* < 0.05) the molar proportion of acetate (−5%) and the A:P ratio (−18%), and increased (*p* < 0.05) the propionate proportion (+16%) when compared with control. Treatment 3 only at 400 mg/L decreased (*p* < 0.05) the molar proportion of acetate (−4%) and the A:P ratio (−17%), and increased (*p* < 0.05) the molar proportion of propionate (+15%) compared with control. Treatment 4 had no effect at 200 mg/L but at 400 mg/L decreased (*p* < 0.05) the molar proportion of acetate (-6%) and the A:P ratio (−25%), and increased (*p* < 0.05) the molar proportion of propionate (+24%) compared with control. Treatment 5 at 200 and 400 mg/L decreased (*p* < 0.05) the molar proportion of acetate (−6 to −8%) and the A:P ratio (−22 to +26%), and increased (*p* < 0.05) the molar proportion of propionate (+21 to +25 %) when compared with control. Treatment 6 had no effect at 200 mg/L, and only the molar proportion of acetate decreased (−5%; *p* < 0.05) at 400 mg/L compared with the control. When mixes modified rumen microbial fermentation (T1 to T6), the relative effects of the mixes of EO were compared with the closest doses of TeTr oil in Experiment 1, and the size of the effect increased, suggesting no additive or synergistic effects.

## 4. Discussion

In the last two decades, there has been a wealth of data produced by research on the effect of EO on rumen microbial fermentation, most of it in in vitro conditions [1,2]. However, most of it has focused on dairy cattle, where fermentation was conducted in high forage diets at pH 6.5–7.0. There are few reports on the effects of EO on ruminal microbial fermentation in high concentrate diets fed to feedlot beef cattle [5,13,14]. The antimicrobial activity of EO is affected by pH, and ruminal pH in cattle fed high-concentrate diets is different from that of dairy cattle diets and often below 6.0 [5]. These effects may also affect potential interactions among different EO in mixes. The selection criteria to identify EO with positive effects on rumen microbial fermentation in these conditions were an increase or no change in total VFA, an increase in the proportion of propionate, and/or a decrease in the proportion of acetate, the A:P ratio and/or the ammonia-N concentration. These effects would improve the efficiency of energy and N utilization [1]. On the other hand, Calsamiglia et al. [1] suggested that, because of different mechanisms of action, the combination of EO may result in additive or synergistic effects. In fact, most EO products in the market are based on combination of different EO or their active molecules (i.e., CRINA-Ruminants^®^, DSM, Switzerland; AGOLIN^®^, Agolin Sa, Switzerland, XTRACT^®^, Pancosma, Switzerland), but scientific evidence of their additive or synergistic effects is lacking. 

Monensin has been recognized as an effective in-feed antibiotic that results in an increase in propionate at the expenses of acetate, and reduces amino acid deamination [15,16] and was used as a positive control. The effects of monensin in Experiments 1 and 2 show these effects and confirm the reliability of the method to measure these changes in the selected EO. At similar concentrations as monensin, EO in Experiment 1 and EO mixes in Experiment 2 had no effects on rumen fermentation, even when considering the concentration of active components. This observation indicates that the activity of EO is weaker compared with monensin, and higher doses are required to provoke a change in rumen microbial fermentation.

### 4.1. Experiment 1 (Individual Essential Oils) 

Ammonia-N was only reduced in Thy and Sag at the highest dose, and the magnitude of the reduction was much lower than that observed for monensin. The effect of Thy on ammonia-N concentration agrees with previous reports where Thy was tested in similar conditions and doses [17]. Because Sag had negative effects on VFA and Thy did not affect VFA at the high dose, the effects of EO on ammonia-N could not be considered as a criterion to select EO for Experiment 2. 

Thyme and Ore contain high concentrations of thymol and carvacrol as major active components, respectively. Thymol and carvacrol are phenolic monoterpenes with a wide-spectrum antimicrobial activity due to its capacity to act as membrane permeabilizer [18,19,20]. Juven et al. [21] observed that their antibacterial activity was greater at pH 5.5 compared with the activity at pH 6.5. In the beef-type diet conditions of this experiment, Ore and Thy at 50 and 200 mg/L were affected in the same way as total and individual VFA, reflecting a similar mechanism of action, and are in agreement with previous results in similar conditions [5,17]. The lack or negative effect of these EO up to 500 mg/L also agrees with previous reports [22,23], but higher doses inhibit rumen microbial fermentation. These results indicate that Ore and Thy should be included at moderate doses to modify rumen fermentation profile without inhibiting microbial activity. Because phenolic compounds with similar structure may have additive effects [6], these two EO were selected for further exploration of possible interactions in Experiment 2. 

Terpinen-4-ol is the main active component in TeTr and has a wide-spectrum antimicrobial activity [24]. The TeTr at 50 and 200 mg/L decreased the molar proportion of acetate and the A:P ratio and increased the molar proportion of propionate and the molar proportion of butyrate, a fermentation profile consistent with better energy utilization [1]. In fact, at 200 mg/L, the effect on the A:P ratio was the largest among all EO tested in Experiment 1. Results agree with Castillejos et al. [22] using similar fermentation conditions and doses. Although some reports have shown negative effects of TeTr on rumen microbial fermentation [25,26,27], with a significant reduction in total VFA and propionate concentrations, these trials were carried out with ruminal liquid and conditions for dairy cows and at pH > 6.0. Several authors have already recognized the role of media pH and type of diet on the antimicrobial activity of EO [5,21]. Therefore, results obtained in the present report with high concentrate beef-type fermentation conditions justify the selection of TeTr for further study in Experiment 2.

Clove bud oil at 200 mg/L increased the molar proportion of butyrate, and at 400 mg/l decreased the molar proportion of propionate and increased the molar proportion of butyrate, the A:P ratio and the pH compared with the control. Castillejos et al. [22] also reported that Clo at 500 mg/L reduced the molar proportion of propionate and increased the molar proportion of butyrate and the A:P ratio. This fermentation profile is not adequate to improve energy utilization in beef cattle diets. In the present trial, Clo at moderate dose (10 and 50 mg/L) increased the ammonia-N concentrations in 1% and 2% vs. control, but differences were not significant. In contrast, at lower doses (30 mg/L), Cardozo et al. [5] reported that eugenol increased total VFA by 20% and increased the concentration of ammonia-N, which could be desirable if ammonia-N concentration limits microbial protein synthesis in feedlot cattle fed high percentages of concentrate [28]. 

Rosemary oil only had effects at the highest dose but reduced total VFA concentrations and the proportion of butyrate, and increased the acetate proportion. This fermentation profile is not desirable in beef cattle conditions, where an increase in propionate is desired [1]. Sage oil at the highest dose decreased the A:P ratio, but at the same time total VFA was also reduced, suggesting that microbial fermentation was inhibited. It is interesting to observe that the active components of Ros and Sag are 1,8-cineole and thujone, respectively, and both are oxygenated phenols. Oxygenated phenols have been reported to have negative or even antagonistic effects when mixed with other phenolic compounds [6], and therefore, were discarded for Experiment 2.

### 4.2. Experiment 2 (Combination of Essential Oils)

In Experiment 1, TeTr, Ore and Thy at 50 and 200 mg/L had the most appropriate fermentation profile to improve energy utilization in beef cattle diets. The main active components of these EO are phenolic monoterpens. Bassole and Juliani [6] indicated that phenolic compounds tend to have additive effects, while synergistic or antagonistic effects would occur with other chemical compounds and vary depending on the microbial ecosystem. Clove bud oil was selected because previous research suggested that at pH 5.5 and at moderate doses stimulated deamination activity and inhibited peptide lyses [3,5]. Because of the different mechanisms of action involved, synergies may be expected. Furthermore, the main active component, eugenol, is a phenylpropanoid, which may have synergies or antagonistic effects with phenolic monoterpenes [6]. There are no previous reports designed to explore interactions between the selected EO on rumen fermentation profile in beef feedlot-type fermentation conditions. 

In Experiment 1, TeTr was the EO with the strongest response at doses of 50 to 200 mg/L. For this reason, most of the mixes proposed contained TeTr at different effective concentrations. Treatments at 200 or 400 mg/L and with 50 or 75% of TeTr (corresponding to an effective dose of TeTr of 100 to 300 mg/L) reduced the proportion of acetate and the A:P ratio, and increased the proportion of propionate without affecting the total concentration of VFA (T1 to T5). The effective doses of TeTr in the mixes were within the range of those tested in Experiment 1, and the magnitude of the response was never higher that the effect observed when TeTr oils was supplemented alone in Experiment 1. Therefore, there is no evidence that the combination of different EO had an additive or synergistic effect neither with monoterpenes, nor with phenolpropanoids. It is also interesting to observe that whenever Ore, Thy or both were mixed with Clo (see T7 to T13, Table 1), the fermentation profile was not affected. In treatments T7 to T13 (Table 1), 100 mg of Thy, Ore or the combination of both resulted in no effect if mixed with 100, 200 or 300 mg of Clo. This is surprising because both Thy and Ore reduced acetate, increase propionate and reduced the A:P ratio when incubated alone in Experiment 1, but this effect was inhibited by the presence of Clo. Treatment T6 contained TeTr, and even in cases where the effective concentration of TeTr was 100 and 200 mg/L that resulted in improved fermentation profile in Experiment 1, there was only a small reduction in the acetate proportion (−5%), no effect on propionate and a non-significant reduction of the A:P ratio. There seem to be an antagonistic effect by which the combination of either Thy, Ore or both with Clo resulted in no effect on rumen fermentation profile. 

None of the mixes in Experiment 2 affected ammonia-N concentration or BCVFA, suggesting that deamination was not affected. This is not surprising, because only Thy and Sag at 400 mg in Experiment 1 had effects, and in none of the treatments in Experiment 2 the effective dose of these EO was reached. Clove bud oil was selected because Busquet et al. [27] indicated that it reduced peptide degradation. We hypothesized that the combination of the inhibition of peptide degradation by eugenol in Clo may be synergistic with inhibition of deaminnation shown in Thy. However, the combination of different EO appear no to have this additive or synergistic effect in protein degradation. These results question the common practice of mixing different EO based on potential additive or synergistic effects.

## 5. Conclusions

Tea tree oil, oregano oil and thyme oil can be effective as rumen fermentation modifiers under feedlot beef-type fermentation conditions. The combinations of TeTr oil with Ore oil, Thy oil or Ore + Thy did not have additive effects over those observed with TeTr oil alone. When Thy, Ore or Thy + Ore where mixed with Clo, all effects on rumen fermentation profile disappeared even when TeTr was part of the mix, suggesting an antagonistic interaction of Clo with Thy and Ore. Results do not support the hypothesis of additivity among the EO tested, and antagonistic effects of Clo mixed with Thy or Ore were demonstrated at least in a low pH, beef-type fermentation conditions.

## Figures and Tables

**Table 1:** Treatments and combination of essential oils evaluated in *in vitro* fermentation.

Treatment Number	Combination of Essential Oils (%)			
	Tea-tree	Clove Bud	Thyme	Oregano
1	75	25	-	-
2	75	-	25	-
3	75	-	-	25
4	50	50	-	-
5	50	-	25	25
6	50	25	25	-
7	50	25	-	25
8	25	50	25	-
9	25	50	-	25
10	25	75	-	-
11	-	75	25	-
12	-	75	-	25
13	-	50	25	25

**Table 2:** Effect of Tea Tree (TeTr), Oregano (Ore), Clove bud (Clo), Thyme (Thy), Rosmarinus officinalis (Ros), Sage oil (Sag) and Monensin (MON) on pH, ammonia-N and total and individual VFA concentrations in vitro fermentation of 10:90 forage to concentrate diet (*n* = 6).

Treatment	pH	NH_3_, mg/dL	Total VFA, mM	Acetate, %	Propionate, %	Butyrate, %	Valerate, %	BCVFA ^2^, mM	A:P ^3^
CTR	5.33	13.2	133.2	61.8	18.8	13.4	3.46	3.47	3.28
TeTr10 ^a^	5.32	13.2	132.7	61.3	18.1	14.4	3.53	3.41	3.38
TeTr50 ^b^	5.33	13.2	129.0	56.5 *	22.2 *	15.2 *	3.56	3.26	2.55 *
TeTr200 ^c^	5.33	13.6	127.0	52.4 *	25.2 *	16.2 *	3.53	3.54	2.11 *
TeTr400 ^d^	5.38	13.2	127.8	60.2	18.3	15.2 *	3.81	3.15	3.28
Ore10 ^a^	5.33	13.7	135.9	59.9	19.9	14.4	3.36	3.32	3.07
Ore50 ^b^	5.33	13.5	132.6	56.0 *	23.3 *	15.0 *	3.25	3.29	2.43 *
Ore200 ^c^	5.33	13.6	128.7	54.8 *	23.5 *	15.5 *	3.53	3.32	2.33 *
Ore400 ^d^	5.44 *	13.6	131.1	60.8	17.6	15.6 *	3.49	3.19	3.44
Clo10 ^a^	5.31	13.3	135.9	60.6	19.0	14.6	3.23	3.33	3.19
Clo50 ^b^	5.33	13.5	133.0	60.9	18.8	14.6	3.12	3.40	3.24
Clo200 ^c^	5.33	13.5	133.1	60.3	19.2	14.7 *	3.22	3.41	3.14
Clo400 ^d^	5.44 *	13.9	127.2	61.8	16.4 *	15.8 *	3.46	3.22	3.77 *
MON	5.44 *	11.1 *	130.0	53.9 *	28.2 *	12.5	3.39	2.64 *	1.99 *
SEM ^1^	0.02	0.80	2.63	0.95	0.83	0.28	0.14	0.20	0.10
CTR	5.26	17.4	131.5	61.8	20.0	14.3	1.60	2.90	3.14
Thy10 ^a^	5.13	17.3	130.1	63.1	20.4	13.0	1.46	2.48	3.14
Thy50 ^b^	5.18	17.9	127.0	58.4 *	24.8 *	13.3	1.49	2.59	2.37 *
Thy200 ^c^	5.25	16.8	127.0	58.3 *	26.0 *	12.2 *	1.37	2.59	2.26 *
Thy400 ^d^	5.37	14.4 *	125.7 *	64.9 *	20.0	11.2 *	1.59	2.44 *	3.30
Ros10 ^a^	5.16	16.9	128.2	63.1	20.6	12.9	1.44	2.47	3.11
Ros50 ^b^	5.15	17.1	128.0	63.1	20.4	12.9	1.66	2.59	3.14
Ros200 ^c^	5.31	17.2	127.1	63.7	20.6	12.3 *	1.39	2.56	3.17
Ros400 ^d^	5.31	17.4	125.3 *	64.3 *	20.2	11.6 *	1.49	2.85	3.30
Sag10 ^a^	5.22	17.4	128.2	63.7	20.0	12.4	1.37	3.28	3.11
Sag50 ^b^	5.19	17.3	129.3	63.7	20.4	12.3	1.37	3.31	3.14
Sag200 ^c^	5.13	17.4	127.5	63.7	20.8	11.8 *	1.33	3.34	3.08
Sag400 ^d^	5.16	14.5 *	125.9 *	60.7	22.4 *	12.9	1.43	3.16	2.72 *
MON	5.24	11.3 *	134.1	55.9 *	25.8 *	14.6	1.73	2.84	2.20 *
SEM ^1^	0.05	0.71	2.00	0.71	0.44	0.70	0.14	0.16	0.08

^1^ SEM—standard error of the mean. ^2^ BCVFA—Branch-chained VFA. ^3^A:P—Acetate to propionate ratio. * Means within a column differ from control (*p* < 0.05). ^a^ 10 mg/L. ^b^ 50 mg/L. ^c^ 200 mg/L. ^d^ 400 mg/L.

**Table 3:** Effect of different doses of Treatment 1 (T1: 75% Tea tree, 25% Thyme), Treatment 2 (T2: 75% Tea tree, 25% Clove bud), Tretament 3 (T3: 75% Tea tree, 25% Oregano), Treatment 4 (T4; 50% Tea tree, 50% Clove bud), Treatment 5 (T5: 50% Tea tree, 25% Thyme, 25% Oregano), Treatment 6 (T6: 50% Tea tree, 25% Clove bud, 25% Thyme) and Monensin (MON) on pH, ammonia-N and total and individual VFA concentrations in vitro fermentation of 10:90 forage to concentrate diet (*n* = 6).

Treatment	pH	NH_3_, mg/dL	VFA, mM	Acetate, %	Propionate, %	Butyrate, %	Valerate, %	BCVFA ^2^, mM	A:P ^3^
CTR	5.33	27.5	130.8	62.0	21.0	14.0	1.08	2.47	2.94
T1 (10) ^a^	5.32	25.2	129.1	61.5	21.3	14.3	1.05	2.54	2.91
T1 (50) ^b^	5.30	25.2	128.2	61.2	21.3	14.7	1.10	2.41	2.87
T1 (200) ^c^	5.30	26.5	128.6	58.6 *	23.6 *	14.3	1.20	2.36	2.48 *
T1 (400) ^d^	5.28+	25.2	126.5	58.3 *	24.4 *	14.4	1.14	2.40	2.39 *
MON	5.29+	25.2 *	123.5 +	58.9 *	25.9 *	12.2 +	1.29	2.10 *	2.28 *
SEM ^1^	0.01	1.34	2.70	0.68	0.53	0.51	0.09	0.05	0.09
CTR	5.38	27.0	116.9	60.1	20.8	15.4	1.24	2.64	2.89
T2 (10) ^a^	5.39	24.9	116.7	60.1	21.5	14.6	1.30	2.69	2.82
T2 (50) ^b^	5.36	26.3	116.3	59.6	21.5	15.6	1.19	2.58	2.80
T2 (200) ^c^	5.38	26.1	114.6	59.6	21.7	15.0	1.38	2.60	2.75 *
T2 (400) ^d^	5.37	27.0	115.0	56.9 *	24.2 *	15.4	1.36	2.59	2.36 *
T3 (10) ^a^	5.36	27.3	115.7	60.0	20.6	15.6	1.20	2.64	2.88
T3 (50) ^b^	5.38	26.6	116.2	59.7	21.5	15.3	1.33	2.62	2.78
T3 (200) ^c^	5.36	26.0	114.9	59.3	21.5	14.8	1.16	2.59	2.81
T3 (400) ^d^	5.37	26.7	116.5	57.3 *	24.0 *	15.1	1.48	2.58	2.40 *
MON	5.36	22.7	114.2	57.5 *	26.5 *	2.03 *	0.98	2.29 *	2.17 *
SEM^1^	0.01	1.34	2.70	0.68	0.53	0.51	0.09	0.05	0.09
CTR	5.40	24.9	126.0	63.8	18.1	14.6	1.09	2.95	3.53
T4 (10) ^a^	5.39	25.6	127.2	62.7	19.0	14.7	1.10	3.01	3.30
T4 (50) ^b^	5.34	25.9	127.2	62.3	19.6	14.5	1.26	3.04	3.19
T4 (200) ^c^	5.38	24.9	127.2	63.2	18.7	14.6	1.18	2.90	3.41
T4 (400) ^d^	5.31	23.0	125.3	59.8 *	22.5 *	14.1	1.14	3.10	2.66 *
T5 (10) ^a^	5.37	24.7	124.9	62.5	19.6	14.6	1.11	2.74	3.20
T5 (50) ^b^	5.33	25.1	127.2	63.0	18.7	14.9	1.14	2.84	3.37
T5 (200) ^c^	5.38	24.2	125.6	60.2 *	21.9 *	14.1	1.28	2.98	2.75 *
T5 (400) ^d^	5.35	21.4	127.2	58.8 *	22.7 *	15.2	1.20	2.93	2.60 *
T6 (10) ^a^	5.41	27.6	129.2	61.6	20.6	14.9	1.22	2.28	3.02
T6 (50) ^b^	5.37	27.7	125.9	61.1	20.8	14.1	1.20	2.37	3.05
T6 (200) ^c^	5.39	28.1	128.6	62.1	21.2	14.7	1.21	2.34	2.92
T6 (400) ^d^	5.38	27.1	127.2	59.3 *	21.0	14.6	1.28	2.39	2.58
MON	5.37	24.1 *	123.5 +	59.6 *	25.1 *	12.3 +	1.31	2.03 *	2.38 *
SEM ^1^	0.01	1.12	2.70	0.90	0.69	0.48	0.10	0.08	0.13

^1^ SEM—standard error of the mean. ^2^ BCVFA—Branch-chained VFA. ^3^ A:P—Acetate to propionate ratio. * Means within a column differ from control (*p* < 0.05). ^a^ 10 mg/L. ^b^ 50 mg/L. ^c^ 200 mg/L. ^d^ 400 mg/L.
